# The roles of circular RNAs in nerve injury and repair

**DOI:** 10.3389/fnmol.2024.1419520

**Published:** 2024-07-15

**Authors:** Ying Zong, Yuqi Dai, Junjie Yan, Bin Yu, Dong Wang, Susu Mao

**Affiliations:** Key Laboratory of Neuroregeneration of Jiangsu and Ministry of Education, School of Medicine, Co-innovation Center of Neuroregeneration, Nantong University, Nantong, China

**Keywords:** circular RNA, nerve regeneration, peripheral nerve injury, traumatic brain injury, spinal cord injury, neuropathic pain

## Abstract

Nerve injuries significantly impact the quality of life for patients, with severe cases posing life-threatening risks. A comprehensive understanding of the pathophysiological mechanisms underlying nerve injury is crucial to the development of effective strategies to promote nerve regeneration. Circular RNAs (circRNAs), a recently characterized class of RNAs distinguished by their covalently closed-loop structures, have been shown to play an important role in various biological processes. Numerous studies have highlighted the pivotal role of circRNAs in nerve regeneration, identifying them as potential therapeutic targets. This review aims to succinctly outline the latest advances in the role of circRNAs related to nerve injury repair and the underlying mechanisms, including peripheral nerve injury, traumatic brain injury, spinal cord injury, and neuropathic pain. Finally, we discuss the potential applications of circRNAs in drug development and consider the potential directions for future research in this field to provide insights into circRNAs in nerve injury repair.

## 1 Introduction

In clinical practice, injuries to the central and peripheral nervous systems are a frequent occurrence. Such neurological injuries, encompassing peripheral nerve injury (PNI), traumatic brain injury (TBI), spinal cord injury (SCI), and neuropathic pain (NP), not only detrimentally impact patient quality of life, but bring substantial economic burdens to families and society at large. However, the current therapeutic options for these conditions are notably limited, largely due to an inadequate understanding of the molecular changes following these injuries.

Circular RNA (circRNA) is a type of covalently closed single-stranded RNA, generated through back-splicing events during canonical RNA splicing, and is ubiquitously present in eukaryotic cells (Aufiero et al., 2019; Liu and Chen, 2022). Remarkable progress in circRNA research have been witnessed in recent years, revealing its pivotal roles in both physiological and pathological processes (Kristensen et al., 2022). The expression and function of circRNAs are subject to stringent regulation, and their expression levels are closely tied to cell type, intracellular signaling cascades, and environmental cues. Furthermore, circRNAs exhibit tissue-specific and cell-specific expression patterns, and their biogenesis is governed by specific cis- and trans-acting elements (Yang et al., 2021). A growing body of evidence highlights the diverse regulatory functions of circRNAs, including the roles as miRNA sponges, anchors for circRNA binding proteins (cRBPs), transcriptional regulators, molecular scaffolds, and sources for translation of small proteins/peptides (Huang et al., 2020; Misir et al., 2022). Notably, dysregulated circRNA expression has been implicated in a variety of diseases, including central nervous system (CNS) disorders, tumors, cardiovascular pathologies, and metabolic disorders, etc. (Yang et al., 2021; Fan et al., 2022; Zhang et al., 2022). Furthermore, circRNAs exhibit remarkable stability in both intracellular and extracellular environments (Liu and Chen, 2022). Consequently, they have emerged as promising biomarkers for the diagnosis of various diseases. In recent years, there has been a growing interest in the study of circRNAs in the context of nerve injury. Evidence suggests that circRNAs may have the potential to ameliorate acute CNS injuries through diverse molecules and pathways. After peripheral nerve injuries, circRNAs have been shown to promote the repair of neurological injuries by facilitating axon regeneration, promoting Schwann cell (SC) proliferation, and regulating neuronal autophagy (Zhou et al., 2018; Sohn and Park, 2020; Liu et al., 2022). Thus, circRNAs hold promise for the treatment of nerve injuries. This review aims to summarize recent advances in circRNA expression profiles (Supplementary Table S1) and roles (Tables 1–4) in this field and present a prospective view of circRNA in the treatment of nerve injury.

**Table 1:** Functional characterization of the circRNAs post peripheral nerve injury (PNI) and the underlying mechanisms.

Model	CircRNA	Regulation	Function	Mechanism	References
PNI	CircRNA 012412	Up	Focal adhesion and endocytosis	CircRNA012412/microRNA/mRNA network	Sohn and Park (2020)
PNI	Circ-Ankib1	Down	Inhibiting SC proliferation and nerve regeneration	DHX9-circ-Ankib1-miR-423-5p/miR-485-5p/miR-666-3p-Cyp26b1 pathway	Mao et al. (2019b)
PNI	Circ-Spidr	Up	Promoting axon regeneration of DRG neurons	Regulating PI3K-Akt signaling pathway	Mao et al. (2019a)
PNI	CircRNA.2837	Down	Regulating autophagy in neurons	As a miRNA sponge for the miR-34 family	Zhou et al. (2018)

**Table 2:** Functional characterization of the circRNAs post traumatic brain injury (TBI) and the underlying mechanisms.

Model	CircRNA	Regulation	Function	Mechanism	References
TBI	CircMETTL9	Up	Promoting neuroinflammation	Binding SND1 and upregulating SND1 expression, leading to the upregulation of pro-inflammatory chemokines in astrocytes	Huang et al. (2023)
TBI	Circ-Scmh1	Down	Promoting microglial M2 polarization	Circ-Scmh1-miR-154-5p-STAT6	Chen et al. (2023)
TBI	CircHtra1	Up	Inhibiting cell proliferation and promoting apoptosis	CircHtra1/miR-3960/GRB10 axis	Zheng et al. (2022b)
TBI	Circ_0116449	Down	Reducing the apoptosis rate and ROS production	Circ_0116449-miR-142-3p-NR1D2 axis	Zheng et al. (2022a)
TBI	CircPtpn14		Promoting ferroptosis	CircPtpn14/miR-351-5p/5-LOX axis	Wu et al. (2022)
TBI	CircRNA_009194	Up	Increasing TBI-induced neurological dysfunctions	CircRNA_009194/miR-145-3p/Nav1.3	Huang et al. (2022)
TBI	CircIgfbp2	Up	Increasing mitochondrial dysfunction and oxidative stress-induced synapse dysfunction	CircIgfbp2/miR-370-3p/BACH1/HO-1 axis	Du et al. (2022)
TBI	CircLrp1b	Up	Increasing TBI-induced autophagy and inflammation	CircLrp1b/miR-27a-3p/Dram2 signaling pathway	Li H. et al. (2020)
TBI	CircRNA_16895	Down	Regulating fragment crystallizable gamma receptor (FccR)-mediated phagocytosis pathway	(Predicted) CircRNA_16895–miRNA–Myo10	Jiang et al. (2019)
TBI	CircRNAchr8_87,859,283-87,904,548	Up	Promoting neuroinflammation	CircRNAchr8_87,859,283–87,904,548-mmu-let-7a-5p-CXCR2	Chen et al. (2019)

**Table 3:** Functional characterization of the circRNAs post spinal cord injury (SCI) and the underlying mechanisms.

Model	CircRNA	Regulation	Function	Mechanism	References
SCI	Circ_0013613	Up	Promoting cytotoxicity, inflammation and inflammation-induced pyroptosis	Circ_0013613/miR-370-3p/CASP1 axis	Guo et al. (2023)
SCI	CircPrkcsh	Up	Promoting the inflammatory response	CircPrkcsh/miR-488/CCL2 axis	Chen et al. (2022)
SCI	CircPlek	Up	Promoting fibrosis activation	CircPlek/miR-135b-5p/TGF-βR1 axis	Wang W. et al. (2021)
SCI	Circ-HIPK3	Down	Reducing the neuronal cell apoptosis	Circ-HIPK3/miR-558/DPYSL5 axis	Zhao et al. (2020)
SCI	Circ 0000962	Down	Reducing nerve cell inflammation	Circ 0000962-miR-302b-3p-PIK3CA/Akt/NF-κB axis	He et al. (2020)
SCI	Circ_006573	Up	Increasing cell apoptosis	Circ_006573/miR-376b-3p axis	Wang K. et al. (2023)
SCI	CircSmox	Up	Contributing to neuroinflammation and apoptosis in PC12 cells	CircSmox/miR-340-5p/Smurf1 axis	Han et al. (2023)
SCI	RNO_CIRCpedia_4,214	Up	Inhibiting macrophage M2-like polarization	RNO_CIRCpedia_4,214/RNO-miR-667-5p/Msr1 axis	Cao et al. (2023)
SCI	Hsa_circ_0026646	Up	Exerting a neuroprotective effect (speculate)	Hsa_circ_0026646/miR-331-3p/*PLXNB2* network	Zu et al. (2022)
SCI	CircHIPK3	Down	Reducing inflammatory response and cell apoptosis	CircHIPK3/miR-382-5p/DUSP1 pathway	Yin et al. (2022)
SCI	Rno-circRNA-013017	Down	Inhibiting apoptosis of motor neurons in the spinal anterior horn	Rno_circRNA_013017/rno-miR-16-5p/bcl-2 pathway (to be verified)	Qin et al. (2022)
SCI	Circ-Ctnnb1	Up	Reducing neuronal apoptosis	Circ-Ctnnb1/miR-205-5p/Ctnnb1/Wnt2b axis	Qi et al. (2022)
SCI	Circ-Ncam2	Up	Promoting microglia activation and neuronal apoptosis	Circ-Ncam2/miR-544-3p/TLR4/NF-κB pathway.	Guo et al. (2022)
SCI	CircRNA_014301	Up	Promoting apoptosis and inflammation	Unclear	Xie et al. (2021)
SCI	CircAbca1	Up	Neuroinhibition	CircAbca1/miR-135b-5p/KLF4 axis	Wang W. Z. et al. (2021)
SCI	Circ-Usp10	Up	Promoting microglial activation and inducing neuronal death	Circ-Usp10/miR-152-5p/CD84	Tong et al. (2021)
SCI	CircTYW1	Down	Suppressing cell apoptosis	CircTYW1/miR-380/FGF9/ERK1/2 axis	Sun et al. (2021)
SCI	Circ_HIPK3	Down	Accelerating cell cycle progression, promoting cell proliferation and inhibiting cell apoptosis	Circ_HIPK3/miR-222-3p/DUSP19 axis	Liu Y. et al. (2021)
SCI	CircPrkcsh	Up	Promoting microglia M1 polarization	CircPrkcsh/miR-488/Mekk1 axis	Li X. et al. (2021)
SCI	CircRNA-2960	Up	Exacerbating the inflammatory response and inducing apoptosis at the lesion site	CircRNA_2,960/miRNA_124	Chen et al. (2021)
SCI	CicRNA.7079	Up	Anti-apoptotic	CicRNA_7,079/mmu-miR-6953-5p/Lgals3 axis	Yao et al. (2020)
SCI	Circ 0001723	Down	Anti-inflammation	Circ 0001723/miR-380-3p-HIF-1α	Li X. et al. (2020)
SCI	CircRNA_01477	Down	Regulator of glial proliferation and migration	CircRNA_01477/miR-423-5p	Wu et al. (2019)
SCI	CircRNA_01477	Down	Decreasing axonal length	CircRNA_01477/miRNA-3075/FosB/Stat3 axis	Qian et al. (2022)
SCI	CircRNA Rims2	Down	Promoting neurite outgrowth	CircRNA Rims2/miRNA (miR-192 or − 377)/GAP-43 mRNA	Siddiq et al. (2023)

**Table 4:** Functional characterization of the circRNAs post neuropathic pain (NP) and the underlying mechanisms.

Model	CircRNA	Regulation	Function	Mechanism	References
NP	CircFhit	Up	Enhancing the NK1R+ neurons’ excitability, resulting in NP	Promoting the expression of its parental gene Fhit in cis	Xu et al. (2023)
NP	CiRNA-Kat6b	Down	Mitigating NP	CiRNA-Kat6b/miRNA-26a/Kcnk1 pathway	Xie et al. (2023)
NP	Circ_0005075	Up	Promoting the neuroinflammation	Circ_0005075/miR-151a-3p-/NOTCH2	Zhang et al. (2021)
NP	CircZNF609	Up	Promoting inflammation factors expression	CircZNF609/miR-22-3p/ENO1 axis	Li L. et al. (2020)
NP	ciRS-7	Up	Upregulating the level of autophagy and inflammation	ciRS-7/ miR-135a-5p	Cai et al. (2020)
NP	CircAnks1a	Up	Contributing to central sensitization and behavioral hypersensitivity	Binding directly to the Vegfb promoter CircAnks1a-miR-324-3p- VEGFB	Zhang et al. (2019)
NP	CircSMEK1	Up	Facilitating NP inflammation and microglia M1 polarization	CircSMEK1/miR-216a-5p/TXNIP axis	Xin et al. (2021)

## 2 The roles of circRNAs in peripheral nerve injury

**Figure 1:**
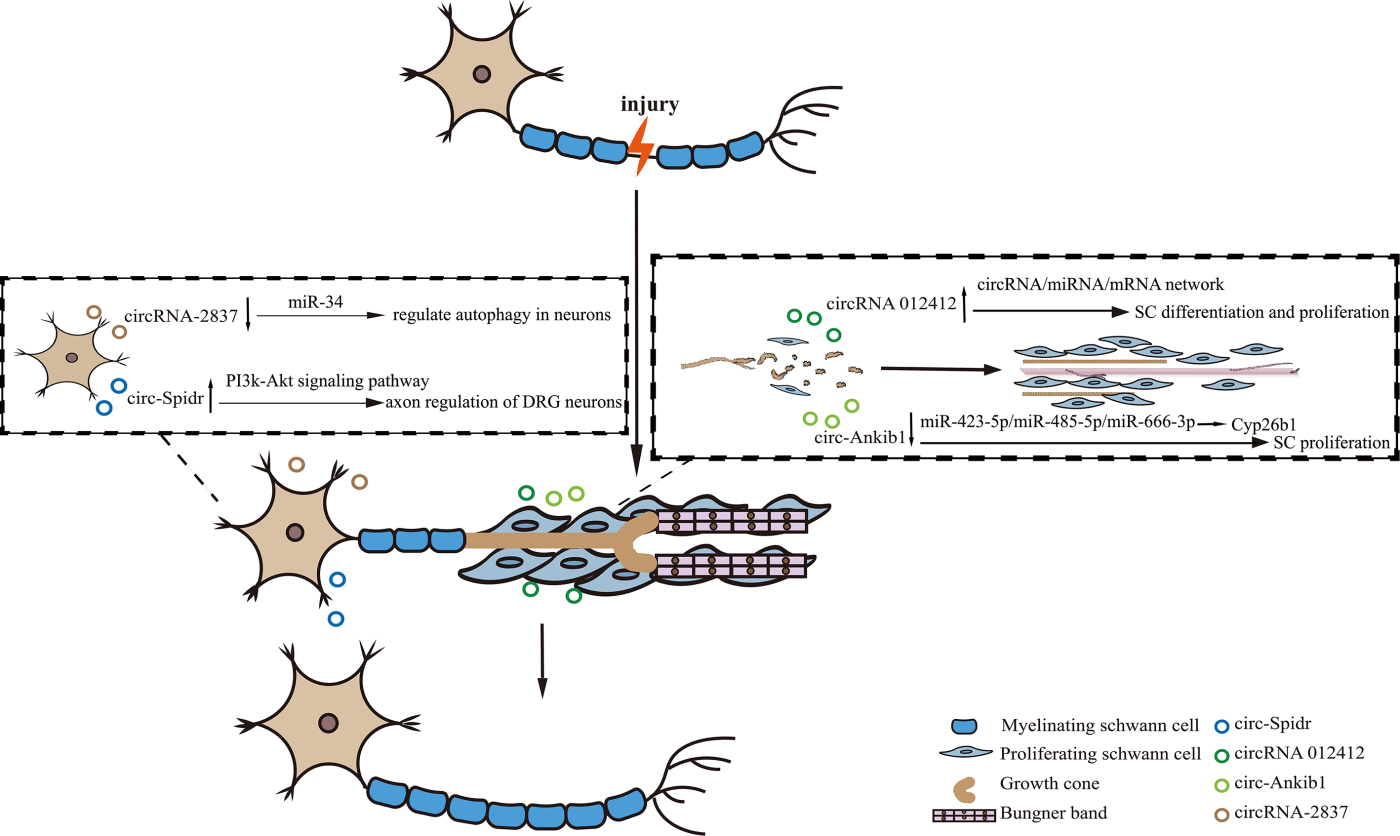
The diagram showing the role of circRNAs in peripheral nerve injury. In the process of peripheral nerve regeneration after injury, circRNAs play crucial roles in neurons or Schwann cells. For instance, circRNA-2837 regulates neuron autophagy, while circ-Spidr enhances axon regeneration. In Schwann cells, circRNA012142 and circ-Ankib1, respectively, regulate cell differentiation and/or proliferation to promote nerve injury repair.

Peripheral nerve injury (PNI) is a condition that results from trauma or disease, affecting nerves outside the CNS, leading to sensory and motor dysfunction. In recent years, there has been an increasing focus on the role of circRNAs in peripheral nerve injury. CircRNAs have been shown to participate in various biological functions during axonal regeneration and degeneration through the circRNA/miRNA/mRNA network. This has been demonstrated by the up-regulation of circRNA 012412 following PNI and the identification of multiple biological functions of the circRNA 012412/microRNA/mRNA network (Sohn and Park, 2020). Our previous work has shown that SCs play a critical role in the repair of nerve injury, and the circRNA circ-Ankib1 is specifically enriched in SCs. After PNI, circ-Ankib1 is down-regulated through the DHX9-circ-Ankib1-miR-423-5p/miR-485-5p/miR-666-3p-Cyp26b1 pathway. This down-regulation induced SC cell proliferation and promoted nerve regeneration (Mao et al., 2019b). Consequently, the targeted modulation of circ-Ankib1 emerges as a promising therapeutic strategy for the enhancement of axonal regeneration after nerve injury. Another circRNA, circ-Spidr, is enriched in the cytoplasm of dorsal root ganglion (DRG) neurons, undergoes upregulation following PNI and contributes to axonal regeneration in these neurons, partially through its regulatory influence on the PI3K-Akt signaling pathway (Mao et al., 2019a). PI3K/Akt pathway was reported to be involved in both neuronal survival and regeneration (Ahn, 2014; Zhang et al., 2014). It suggests that overexpression of circ-Spidr may serve as a viable therapeutic approach for the promotion of axonal regeneration after nerve injury. Additionally, circRNA 2,837 is down-regulated after PNI, and its reduction has been shown to alleviate sciatic nerve injury by inducing autophagy *in vivo*. Knockdown of circRNA 2,837 has been shown to protect neurons from nerve injury by acting as a sponge for members of the miR-34 family members (Zhou et al., 2018). These discoveries underscore the intricate regulatory roles of circRNAs in peripheral nerve injury and hint at their potential therapeutic utility in the context of nerve regeneration (Figure 1).

## 3 The roles of circRNAs in traumatic brain injury

Traumatic brain injury (TBI) refers to brain damage caused by an external force, such as a blow or jolt to the head or penetration of the skull. The immediate aftermath of TBI can manifest as unconsciousness, confusion, memory loss, and neurological deficits, while the enduring effects may include cognitive impairment, motor dysfunction, and psychological disorders. Neuroinflammation plays a crucial role in the cellular response after TBI (Wu et al., 2021). In animal models of TBI, various circRNAs have been linked to neuroinflammation. For example, circMETTL9 has been found to bind to SND1, a multifunctional protein that is involved in regulating neuroinflammation, boosts SND1 expression and elevates the production of pro-inflammatory chemokines in astrocytes. This cascade of events triggers neuroinflammation and neurodegeneration (Huang et al., 2023). In another study, the circRNA circLrp1b was identified as a mediator in the inhibition of Dexmedetomidine in the inflammatory response and autophagy involved in TBI *in vivo* through its interaction with the miR-27a-3p/Dram2 axis (Li H. et al., 2020). Furthermore, the circRNA chr8_87,859,283-87,904,548 was found to be significantly upregulated following TBI, thereby promoting neuroinflammation by increasing CXCR2 protein levels via the sequestration of mmu-let-7a-5p (Chen et al., 2019). Notably, in the serum of mouse with TBI, a down-regulation of circ-Scmh1 was observed in exosomes derived from adipose-derived stem cells (ADSCs). The overexpression of circ-Scmh1 was shown to mitigate inflammation-induced hippocampal nerve injury by inducing the polarization of M2 microglia and suppressing inflammation (Chen et al., 2023). These findings underscore the crucial role of circRNA in the regulation of neuroinflammation after TBI. Moreover, lipid peroxidation has been established as a biomarker for secondary brain injury in both traumatic and non-traumatic contexts (Piastra et al., 2020). Investigating the contributions of circRNAs and lipid peroxidation to the sequelae of TBI could potentially lead to the development of more effective treatments and interventions for those afflicted with this condition. In a study by Zheng et al., 2022a, the down-regulation of circ_0116449 was observed and its role in regulating lipid metabolism in TBI through the circ_0116449-miR-142-3p-NR1D2 axis was elucidated. Another circRNA, circPtpn14, was negatively regulated after melatonin treatment, which exerted anti-ferroptotic and anti-endoplasmic reticulum (ER) stress effects in brain injury by alleviating lipid peroxidation via the circPtpn14/miR-351-5p/5-LOX axis (Wu et al., 2022).

After brain injury, a significant increase in CircHtra1 expression was noted, and this upregulation was correlated with the extent of injury severity. Research revealed that CircHtra1 contributed to neuronal loss by sponging miR-3960 and modulating GRB10 and apoptosis (Zheng et al., 2022b). Nav1.3, one of the large pore α subunit proteins of voltage-gated sodium channels, has been identified as a pivotal voltage sensor in the cell membrane. Notably, it exhibits a significant upregulation in rat’s cortex following TBI, with its expression levels closely linked to the severity of the TBI (Huang et al., 2013). A circRNA circRNA_009194 was observed to be upregulated after TBI. And its suppression, directly or indirectly, suppressed Nav1.3, thereby enhancing neurological outcomes *in vivo* (Huang et al., 2022). Mitochondrial dysfunction and oxidative stress represent pivotal pathological hallmarks in the early stages of TBI. Inhibition of circIgfbp2 was found to alleviate mitochondrial dysfunction and synaptic dysfunction induced by oxidative stress post TBI through the miR-370-3p/BACH1/HO-1 axis (Du et al., 2022). Furthermore, diminished levels of circRNA_16895 post TBI predicted the regulation of the regulatory fragment crystallizable γ receptor (FcγR)-mediated phagocytosis pathway, which is modulated by the circRNA_16895-miRNA-Myo10 axis (Jiang et al., 2019). These discoveries highlight the diverse roles of circRNAs in the pathogenesis of TBI and propose potential therapeutic targets.

## 4 The roles of circRNAs in spinal cord injury

Spinal cord injury (SCI) involves a cascade of biological processes that lead to tissue impairment and neurological dysfunction. The primary injury is precipitated by mechanical trauma, resulting in immediate disruption of neural pathways. Secondary injury mechanisms, including inflammation, excitotoxicity, and oxidative stress, further exacerbate tissue damage and lead to neuronal death. A comprehensive understanding of these processes is imperative for the development of effective therapies to mitigate secondary injury and promote neural repair in SCI. While the functions of certain circRNAs in SCI have been summarized in a previous review (Xu et al., 2021), this review integrates the latest insights into the function of circRNAs in SCI.

### 4.1 CircRNAs in inflammation

In the realm of SCI, inflammation is one of the predominant secondary injuries. The inflammatory cascade post SCI encompasses the accumulation of inflammatory cytokines, activation of microglia, recruitment of macrophages, and invasion of neutrophils (Freyermuth-Trujillo et al., 2022). Various circRNAs have been identified to modulate distinct inflammatory events to promote recovery after SCI, with details provided below.

#### 4.1.1 Inflammatory cytokines

The interplay between inflammatory cytokines and circRNAs has emerged as a pivotal factor in the repair process for SCI. While certain inflammatory cytokines are conducive to spinal cord repair, others can exacerbate damage (Ren et al., 2018). Post-SCI, the surge in pro-inflammatory cytokines can lead to neuronal injury. Notably, specific circRNAs have been implicated in the release of pro-inflammatory cytokines. For instance, the expression of circ_0013613 is elevated following SCI. By knocking down circ_0013613, the cytotoxicity, inflammation, and inflammation-induced cell pyroptosis are mitigated via the miR-370-3p/caspase1 pathway, thus promoting SCI recovery (Guo et al., 2023). In a study by Chen et al. (2022), it was found that inhibiting circPrkcsh reduced the inflammatory response following SCI by upregulating miR-488, which in turn decreased CCL2 level and curtailed inflammatory cytokine secretion *in vitro*. Following SCI, circHecw1 is down-regulated, and its knockdown has been observed to attenuate inflammation in neuronal cells by modulating the miR-3551-3p/LRRTM1 signaling pathway (Ban et al., 2022). Additionally, circ0000962 is down-regulated after SCI, and its overexpression can reduce the inflammatory response through the miR-302b-3p-mediated PI3K/Akt/NF-κB signaling (He et al., 2020). CircSmox expression was also increased after SCI, and its knockdown alleviates LPS-induced neuroinflammation and apoptosis in PC12 cells via the miR-12-340p/Smurf5 axis, exerting a neuroprotective effect (Han et al., 2023). Furthermore, circRNA_014301 is associated with neuroinflammation and its silencing has been shown to attenuate apoptosis and inflammation in PC12 cells (Xie et al., 2021). These intricate interplays between circRNAs and inflammatory pathways highlight the potential for novel therapeutic interventions in the context of SCI.

#### 4.1.2 Microglia and macrophages

After SCI, activated M1-like microglia initiate a cascade of neurotoxic responses, leading to cell apoptosis and necrosis (Freyermuth-Trujillo et al., 2022). In a mouse model of SCI, circ-Ncam2 is found to be up-regulated. The silence of circ-Ncam2 has demonstrated a reduction in LPS-induced microglia activation and neuronal apoptosis by blocking the TLR4/NF-κB pathway, functioning as a miR-544-3p sponge (Guo et al., 2022). Moreover, circ-Usp10, which is up-regulated after SCI, promotes microglia activation by targeting miR-152-5p/CD84 and induces neuronal death (Tong et al., 2021). Another study revealed that circPrkcsh promotes microglia M1 polarization through the miR-488/MEKK1/JNK/p38 MAPK signaling pathway, thus aggravating SCI injury. Consequently, down-regulating circPrkcsh can reduce inflammation and promote recovery from SCI (Li X. et al., 2021).

Macrophages can exert either beneficial or detrimental effects after SCI, depending on their phenotype, corresponding to the classes M1 (pro-inflammatory) and M2 (anti-inflammatory) (Shechter et al., 2013; Van Broeckhoven et al., 2021). Preventing pro-inflammatory macrophage/microglia polarization can promote damage repair. A study has constructed a ceRNA network closely related to SCI immunoinflammation, and the rno_CIRCpedia_4,214/rno-miR-667-5p/Msr1 axis may potentially promote macrophage M2-like polarization in SCI, serving as an effective strategy to mitigate the severity of secondary SCI injury (Cao et al., 2023). During the tissue repair process after SCI, a transmembrane receptor PLXNB2 was reported to induce microglia motility, steer injury-activated microglia/macrophages away from colliding cells, and facilitate matrix compaction (Zhou et al., 2020). Subsequently, Zu et al. proved that hsa_circ_0026646 was upregulated in SCI, which correlates with PLXNB2 by targeting miR-331-3p, providing novel insights into the regulatory mechanisms associated with SCI and potential therapeutic targets for its management (Zu et al., 2022).

### 4.2 CircRNAs in cell apoptosis

Secondary injury following SCI is considered to be due to the continuation of cellular destruction through cell apoptosis. Therefore, the identification of methods to inhibit cell apoptosis after SCI holds significant value in repairing nerve damage (Shi et al., 2021). Circ-HIPK3 has been shown to regulate the biological behavior of neuronal cells. After SCI, the expression of circ-HIPK3 was negatively regulated. However, up-regulation of circ-HIPK3 expression alleviated neuronal cell apoptosis in SCI by regulating the miR-588/DPYSL5 axis (Zhao et al., 2020). Furthermore, it has been shown that overexpression of circHIPK3 reduced the inflammatory response and neuronal apoptosis in AGE1.HN cells induced by oxygen–glucose deprivation, which is achieved by regulating the miR-382-5p/DUSP1 axis (Yin et al., 2022). In another study, circ_HIPK3 was found to alleviate CoCl_2_-induced apoptotic injury in neuronal cells by modulating the miR-222-3p/DUSP19 axis (Liu Y. et al., 2021). SCI inflicts injury on vascular endothelial cells, leading to the leakage of vascular material and accumulation of various inflammatory cytokines, which aggravate the inflammatory reaction (Freyermuth-Trujillo et al., 2022). In a study, circ_006573 was found to be significantly up-regulated after SCI, and the circ_006573/miR-376b-3p axis promoted the recovery of spinal cord function after injury by regulating vascular remodeling (Wang K. et al., 2023). Moreover, rno-circRNA-013017 was significantly down-regulated in rats after injury. Overexpression of rno-circRNA-013017 was found to inhibit apoptosis of motor neurons in the spinal anterior horn 3 days after SCI, thereby contributing to motor function in rats after SCI (Qin et al., 2022). Circ-Ctnnb1 exhibits high expression in both the rat model of SCI and neuronal cells challenged by hypoxia. Its deletion has been observed to elevate the apoptosis rate in neuronal cells challenged by hypoxia. Mechanistically, circ-Ctnnb1 activates the Wnt/β-catenin pathway, a well-known pathway that inhibits apoptosis of neuronal cells (Gao et al., 2016), reversing SCI by sponging miR-205-5p to up-regulate Ctnnb1 and Wnt2b (Qi et al., 2022). Additionally, CircTYW1 expression is negatively regulated after SCI, and circTYW1 has been found to promote neurological recovery in SCI of rats and inhibit apoptosis in spinal cord tissues. CircTYW1 can promote neurological recovery after SCI through the miR-380/FGF9/ERK1/2 regulatory axis. Thus, overexpression of circTYW1 may serve as a novel therapeutic approach to SCI (Sun et al., 2021).

### 4.3 CircRNAs in axonal regeneration

The axonal regeneration of mature neurons is inhibited, while long-distance regeneration of axons plays a critical role in functional recovery after SCI (Alilain et al., 2011; Hilton et al., 2022). Certain circRNAs have been identified as potential players in axonal elongation and repair of SCI. CircRNA_01477 was down-regulated after SCI, and its knockdown significantly increased the axonal length by regulating the miRNA-3075/FosB/Stat3 axis (Qian et al., 2022). Axonal translation is believed to enable neurorepair after SCI. CircRNA Rims2 has been shown to regulate axonal regeneration through miRNAs that modulate the GAP-43 axonal translation, highlighting a crucial role of circRNA in axonal translation in nerve repair (Siddiq et al., 2023). In an inflammatory environment, fibroblasts and astrocytes activation leads to scar formation, which is a significant barrier to axonal regeneration after SCI. CircPlek has been demonstrated to promote fibrotic activation by modulating the miR-135b-5p/TGF-βR1 axis following SCI (Wang W. et al., 2021). In a separate investigation concerning traumatic SCI, silencing circAbca1 is believed to have a neuroprotective effect via the circAbca1/miR-135b-5p/KLF4 network. Within this network, the zinc finger-containing transcription factor KLF4 is implicated in the inhibition of axon regeneration (Qin et al., 2013; Wang W. Z. et al., 2021).

### 4.4 CircRNAs in exosomes

Exosomes derived from mesenchymal stem cells hold great promise for the treatment of SCI (Liu W. Z. et al., 2021). Specifically, hypoxia-induced expression of exosomal circular RNA OXNAD1 (circOXNAD1), derived from human umbilical cord mesenchymal stem cells (HucMSCs), has been demonstrated to alleviate spinal cord ischemia–reperfusion injury in SCI through the circOXNAD1/miR-29a-3p/FOXO3a axis (Wang X. et al., 2023). Moreover, bone marrow mesenchymal stem cells (BMSCs) have exhibited certain therapeutic effects on SCI (Cofano et al., 2019), and BMSC-derived exosomes have been shown to improve recovery from SCI by alleviating inflammasome-associated sepsis by delivering circ_003564 (Zhao Y. et al., 2022). CircZFHX3, also derived from exosomes, has been observed to counteract LPS-induced damage in BV-2 cells, partially by regulating the miR-16-5p/IGF-1 axis, suggesting its potential as a therapeutic strategy for SCI (Tian et al., 2022).

## 5 The roles of circRNAs in neuropathic pain

Neuropathic pain (NP) typically arises from nerve injury, where damage or dysfunction disrupts normal nerve signaling. This chronic pain state can be precipitated by a variety of factors, including trauma, disease, or surgical intervention. Nerve injury triggers abnormal nerve firing, heightened sensitivity, and altered nociceptive perceptions, all of which contribute to enduring discomfort. Additionally, inflammatory responses and biochemical changes further exacerbate pain sensations. Notably, NP frequently persists even after the initial injury has healed, significantly impacting the quality of life. Deciphering the interplay between these factors is crucial for the development of targeted interventions aimed at alleviating NP and improving patient outcomes. A recent study has identified circFhit, an exon-intron circRNA expressed in GABAergic neurons, as a significant contributor to NP by increasing NK1R neuronal hyperexcitation through the regulation of Fhit in cis (Xu et al., 2023). Central sensitization, particularly in the dorsal spinal cord, is considered a central role player in pain hypersensitivity. In NP models, down-regulation of ciRNA-Kat6b in the spinal dorsal horn exacerbated pain hypersensitivity, while blocking this down-regulation attenuated NP through the ciRNA-Kat6b/miRNA-26a/Kcnk1 pathway (Xie et al., 2023). Post-nerve injury, the up-regulation of inflammation-associated circ_0005075 exacerbated pain progression, which can be alleviated by circ_0005075 deletion-induced miR-151a-3p up-regulation and NOTCH2 signaling inactivation (Zhang et al., 2021). Similarly, in a CCI rat model, circZNF609 exacerbated NP via the miR-22-3p/ENO1 axis (Li L. et al., 2020). Moreover, the upregulation of ciRS-7 has been observed to promote NP through interaction with miR-135a-5p, enhancing inflammation and autophagy (Cai et al., 2020). Another up-regulated circRNA in dorsal horn neurons during NP is circAnks1a. The interaction between circAnks1a and YBX1 promotes YBX1 translocation into the nucleus and enhances its binding to the Vegfb promoter, thus promoting Vegfb transcription in dorsal horn neurons after nerve injury. Increased levels of circAnks1a also act as a sponge for miR-324-3p to modulate the translation of Vegfb mRNA in the dorsal horn. Down-regulation of circAnks1a attenuates pain-like behaviors induced by nerve injury (Zhang et al., 2019). Thioredoxin interacting protein (TXNIP) is highly expressed in NP, and usually binds to the NLRP3 inflammasome to affect inflammation, oxidative phosphorylation, and apoptosis to modulate pain. A study found that the expression of TXNIP is mediated by circSMEK1, which is upregulated in NP and induces NP in rats by acting as a sponge for microRNA-216a-5p (Xin et al., 2021). In summary, these findings highlight the multifaceted roles of circRNAs in NP pathways, providing valuable information on potential therapeutic targets for pain management.

## 6 Summary and perspective

This review article offers a comprehensive summary of recent advancements in the roles of circRNAs in nerve injury and repair. The majority of current studies focus on elucidating the involvement of circRNAs in signaling pathways and gene regulatory networks pertinent to nerve injury. Two primary mechanisms have been identified: acting as sponge RNAs and directly binding to proteins. However, circRNAs exhibit multiple other functions, including translating polypeptides, forming pseudogenes, and so on (Misir et al., 2022). Future studies should explore the functions and mechanisms of circRNAs in nerve injury repair based on these alternative functions.

Exosomes derived from mesenchymal stem cells play a crucial role in nerve regeneration after injury. This review also highlights several exosomal circRNAs that contribute to nerve injury repair, particularly in TBI and SCI. However, the role of exosomal circRNAs in peripheral nerve injury and NP requires further investigation. Several studies have shown that Schwann cell-derived exosomes can treat peripheral nerve injury by inhibiting inflammation and promoting axonal regeneration (Qing et al., 2018; Sun et al., 2023). Additionally, exosomes derived from Schwann cell-like cells can also promote nerve injury recovery by facilitating myelin formation and angiogenesis (Hu et al., 2023). However, it remains to be determined whether these exosomes contain circRNAs that promote nerve injury repair, serving as a direction for future research on peripheral nerve injury repair.

Given their greater stability in comparison to linear RNAs, circRNAs are expected to emerge as the preferred RNA platform in the pharmaceutical industry. CircRNA vaccines share numerous similarities with linear mRNA vaccines and hold immense potential as the next generation of RNA-based vaccine platforms (Niu et al., 2023). Compared to mRNA vaccines, circRNA vaccines have the advantage of continuous expression and are less susceptible to degradation (Zhao X. et al., 2022). Consequently, they can generate more durable antigens, thereby eliciting a more robust immune response. Currently, several circRNA-based drugs or vaccines have emerged, such as the SARS-CoV-2 circRNA vaccine, which expresses the spike protein trimer RBD and can effectively stimulate the production of neutralizing antibodies and T-cell responses, providing substantial protection for the body (Qu et al., 2022). Currently, there is no circRNA-based drug reported in repairing human nerve injuries. However, several studies have found that the expression of certain circRNAs is significantly increased in patients with acute ischemic stroke, and their levels positively correlate with infarct volume, suggesting their potential as biomarkers for the diagnosis and prognosis of acute ischemic stroke (Zuo et al., 2020; Li S. et al., 2021). This highlights the important roles circRNAs play in human pathophysiology in the nervous system. Regarding the function of circRNA in nerve injury repair, current research is primarily focused on rodent models. The translational potential of circRNAs identified in these models to human clinical applications remains an important direction for future research. Due to the high conservation of circRNA across species, it is possible that the targets identified in rodents may have significant clinical effects in human as well. Studies have shown that circRNAs exhibit a very high degree of conservation among primates (Santos-Rodriguez et al., 2021). Therefore, further clinical studies or trials of circRNA-based therapeutics could be conducted in non-human primates before advancing to human clinical trials. However, two challenges must be addressed for the future applications of circular RNA drugs or vaccines: the development of efficient production methods and the optimization of delivery systems. Purification of circular RNA products using existing technology is extremely challenging, and effective isolation and purification represent indispensable steps in the production of circular RNA products. The advent of delivery vehicles such as lipid nanoparticles offers hope for the successful delivery of circular RNA (Loan Young et al., 2023). Further investigation addressing these two challenges is elementary to fully explore the potential of applying circRNA-based strategies in clinics. Hopefully, circRNA-based drugs targeting nerve damage repair can be developed soon.

## 7 Conclusion

In conclusion, the investigation of circRNAs in nerve injury remains highly valuable, and more research is needed to uncover new functions and mechanisms of circRNAs in the repair of nerve injury. Furthermore, the development of drugs based on circRNAs holds great promise in paving the way for novel breakthroughs in the treatment of nerve injury and merits further attention.

## Author contributions

YZ: Writing – original draft, Writing – review & editing, Investigation. YD: Investigation, Writing – original draft, Writing – review & editing, Visualization. JY: Investigation, Writing – review & editing. BY: Writing – review & editing, Funding acquisition. DW: Writing – review & editing, Project administration, Supervision. SM: Project administration, Supervision, Writing – review & editing, Conceptualization, Funding acquisition, Writing – original draft.
