# Supplementary material for: The roles of circular RNAs in nerve injury and repair
Source: Front Mol Neurosci. 2024 Jul 15;17:1419520. doi: 10.3389/fnmol.2024.1419520 (PMC11284605; doi:10.3389/fnmol.2024.1419520)
Supplement: Supplementary file 1 [file Table1.DOCX]

Supplementary Material

**Supplementary Table 1.** **Circular RNA (circRNA) expression profiles post nerve injury.**

| **Injury model** | **Tissue** | **Time points post injury** | **Methods** | **References** |
| --- | --- | --- | --- | --- |
| **Peripheral nerve injury** |  |  |  |  |
| Mice sciatic nerve axotomy | Proximal and distal sciatic nerve | 7 d | Microarray | (Sohn and Park, 2020) |
| Rat sciatic nerve crush | Proximal sciatic nerve | 0, 1, 4, 7, and 14 d | RNA-Seq | (Mao et al., 2019b) |
| Rat sciatic nerve crush | (L)4–5 DRGs | 0 h, 3 h, 9 h, 1 d, 4 d and 7 d | RNA-Seq | (Mao et al., 2019a) |
| Rat sciatic nerve compression | Sciatic nerve | 1 d | RNA-Seq | (Zhou et al., 2018) |
| Mice sciatic nerve injury | Gastrocnemius muscles | 0, 1, 2, 4, and 8 w | RNA-Seq | (Weng et al., 2018) |
| **Traumatic brain injury** |  |  |  |  |
| Mice CCI | Brain samples | 3 d | RNA-Seq | (Tan et al., 2024) |
| Rat CCI | Cerebral cortex | 1, 3, 7, and 14 d | Microarray | (Huang et al., 2023) |
| Mice CCI | Exosomes |  | RNA-seq | (Sun et al., 2023) |
| Mice FPI | Exosomes | 3 h | RNA-seq | (Zhao et al., 2018) |
| Mice CCI | Brain | 3 d | RNA-seq | (Zheng et al., 2022) |
| Mice CCI | Brain | 3 d | RNA-seq | (Wu et al., 2022a) |
| Rat FPI | Hippocampus |  | Microarray | (Huang et al., 2022) |
| Mice CCI | Brain samples | 3 d | RNA-seq | (Fu et al., 2022) |
| Mice CCI | Brain tissue |  | RNA-Seq | (Du et al., 2022) |
| Mice CCI | Parietotemporal | 6 h | RNA-Seq | (Jiang et al., 2019) |
| Mice CCI | Brain cortex | 1 d | RNA-Seq | (Chen et al., 2019) |
| Rat FPI | Hippocampus | 3 h | Microarray | (Xie et al., 2018) |
| **Spinal cord injury** |  |  |  |  |
| Rats transection SCI | T10 spinal cord | 7 d | RNA-seq | (Xiao et al., 2022) |
| Mice contusion SCI | T10 spinal cord | 4 d | RNA-seq | (Li et al., 2022) |
| Mice contusion SCI | T8-10 spinal cord | 3 d | RNA-seq | (Chen et al., 2022) |
| Mice contusion SCI | Spinal cord | 1, 3, 7 and 14 d | RNA-seq | (Wang et al., 2021a) |
| Rats contusion SCI | T10 spinal cord |  | RT-qPCR | (Zhao et al., 2020) |
| Rats contusion SCI | T8 spinal cord |  |  | (He et al., 2020) |
| Rats contusion SCI | L2 and L5 spinal cord | 1 d |  | (Wang et al., 2023b) |
| Rats contusion SCI | T10 spinal cord |  | RNA-seq | (Wang et al., 2023a) |
| Rats contusion SCI | T10 spinal cord | 3 d | Microarray | (Cao et al., 2023) |
| Rats contusion SCI | Spinal cord | 3 d | Microarray | (Zu et al., 2022) |
| Rats contusion SCI | Exosomes | 1 d | RNA-seq | (Zan et al., 2022) |
| Rats contusion SCI | T8 spinal cord | 1, 3, 7, 14 d | RNA-seq | (Xu et al., 2022) |
| Rats hemisection SCI | T10 spinal cord | 2 w | RNA-seq | (Wu et al., 2022b) |
| Mice contusion SCI | Exosomes |  |  | (Tian et al., 2022) |
| Rats contusion SCI | T9-11 spinal cord | 3 d | Microarray | (Qin et al., 2022) |
| Rats contusion SCI | T10 spinal cord |  |  | (Qi et al., 2022) |
| Mice contusion SCI | T9-10 spinal cord | 3 d | Microarray | (Ye et al., 2021) |
| Mice contusion SCI | T8-10 spinal cord | 5 d | RNA-seq | (Wang et al., 2021b) |
| Mice contusion SCI | T9 spinal cord | 3 d | RNA-seq | (Tong et al., 2021) |
| Rats contusion SCI | T8 spinal cord |  |  | (Sun et al., 2021) |
| Mice contusion SCI | T8-10 spinal cord | 3 d | RT-qPCR | (Li et al., 2021) |
| Rats contusion SCI | T9-10 spinal cord | 7 d | RNA-seq | (Chen et al., 2021) |
| Mice contusion SCI | T9-10 spinal cord | 3 d | Microarray | (Yao et al., 2020) |
| Mice contusion SCI | T8-10 spinal cord | 3 d | RNA-seq | (Wang et al., 2020) |
| Rats contusion SCI | T10 spinal cord | 2 h | Microarray | (Liu et al., 2020) |
| Rats contusion SCI | T8 spinal cord |  | Microarray | (Li et al., 2020) |
| Rats contusion SCI | T9 spinal cord | 6 h | RNA-seq | (Zhou et al., 2019) |
| Rats contusion SCI | T9 spinal cord | 0, 1, 3, 7, 14, 21, and 28 d | RNA-seq | (Wu et al., 2019) |
| Rats contusion SCI | T10 spinal cord | 3 d | Microarray | (Qin et al., 2018) |
| Rats contusion SCI | T8 spinal cord | 1 d | RNA-seq | (Siddiq et al., 2023) |
| **Neuropathic pain** |  |  |  |  |
| Mice SNT | L3 and L4 DRGs | 3 d | RNA-seq | (Yu et al., 2023) |
| Rats SNI | L4-6 spinal cord |  | RNA-seq | (Xu et al., 2023) |
| Mice CCI | L3-4 DRGs |  | RNA-seq | (Xie et al., 2023) |
| Rats CCI | L4-5 DRGs | 14 d | RNA-seq | (Xiong et al., 2022) |
| Mice CCI-ION | Left TG | 7 d | RNA-seq | (Fang et al., 2022) |
| Rats CCI | L4-6 dorsal spinal cord | 1, 3, 7, 14 and 21 d | RT-qPCR | (Zhang et al., 2021) |
| Rats CCI | L4-5 spinal cord | 0, 3, 7, 11, 15, 20 d |  | (Cai et al., 2020) |
| Rats SNL | Spinal dorsal horn tissue | 7,14 d | RNA-seq | (Zhang et al., 2019) |
| Rats SNI | L4-5 spinal cord | 14 d | RNA-seq | (Zhou et al., 2017) |
| Rats CCI | L3-5 spinal cord | 14 d | Microarray | (Cao et al., 2017) |
| Rats CCI | L4-5 lumbar | 14 d | RT-qPCR | (Xin et al., 2021) |
| Rats CCI | Hippocampus | 21 d | RNA-seq | (Liu et al., 2022) |

**References**

CAI, W., ZHANG, Y. & SU, Z. 2020. ciRS-7 targeting miR-135a-5p promotes neuropathic pain in CCI rats via inflammation and autophagy. *Gene,* 736**,** 144386.

CAO, J., PAN, C., ZHANG, J., CHEN, Q., LI, T., HE, D. & CHENG, X. 2023. Analysis and verification of the circRNA regulatory network RNO_CIRCpedia_ 4214/RNO-miR-667-5p/Msr1 axis as a potential ceRNA promoting macrophage M2-like polarization in spinal cord injury. *BMC Genomics,* 24**,** 181.

CAO, S., DENG, W., LI, Y., QIN, B., ZHANG, L., YU, S., XIE, P., XIAO, Z. & YU, T. 2017. Chronic constriction injury of sciatic nerve changes circular RNA expression in rat spinal dorsal horn. *J Pain Res,* 10**,** 1687-1696.

CHEN, J., FU, B., BAO, J., SU, R., ZHAO, H. & LIU, Z. 2021. Novel circular RNA 2960 contributes to secondary damage of spinal cord injury by sponging miRNA-124. *J Comp Neurol,* 529**,** 1456-1464.

CHEN, J. N., ZHANG, Y. N., TIAN, L. G., ZHANG, Y., LI, X. Y. & NING, B. 2022. Down-regulating Circular RNA Prkcsh suppresses the inflammatory response after spinal cord injury. *Neural Regen Res,* 17**,** 144-151.

CHEN, Z., WANG, H., ZHONG, J., YANG, J., DARWAZEH, R., TIAN, X., HUANG, Z., JIANG, L., CHENG, C., WU, Y., GUO, Z. & SUN, X. 2019. Significant changes in circular RNA in the mouse cerebral cortex around an injury site after traumatic brain injury. *Exp Neurol,* 313**,** 37-48.

DU, M., WU, C., YU, R., CHENG, Y., TANG, Z., WU, B., FU, J., TAN, W., ZHOU, Q., ZHU, Z., BALAWI, E., HUANG, X., MA, J. & LIAO, Z. B. 2022. A novel circular RNA, circIgfbp2, links neural plasticity and anxiety through targeting mitochondrial dysfunction and oxidative stress-induced synapse dysfunction after traumatic brain injury. *Mol Psychiatry,* 27**,** 4575-4589.

FANG, Z. H., LIAO, H. L., TANG, Q. F., LIU, Y. J., ZHANG, Y. Y., LIN, J., YU, H. P., ZHOU, C., LI, C. J., LIU, F. & SHEN, J. F. 2022. Interactions Among Non-Coding RNAs and mRNAs in the Trigeminal Ganglion Associated with Neuropathic Pain. *J Pain Res,* 15**,** 2967-2988.

FU, J., ZHOU, Q., WU, B., HUANG, X., TANG, Z., TAN, W., ZHU, Z., DU, M., WU, C., MA, J., BALAWI, E. & LIAO, Z. B. 2022. Protective effects and regulatory pathways of melatonin in traumatic brain injury mice model: Transcriptomics and bioinformatics analysis. *Front Mol Neurosci,* 15**,** 974060.

HE, R., TANG, G. L., NIU, L., GE, C., ZHANG, X. Q., JI, X. F., FANG, H., LUO, Z. L., CHEN, M. & SHANG, X. F. 2020. Quietness Circ 0000962 promoted nerve cell inflammation through PIK3CA/Akt/NF-κB signaling by miR-302b-3p in spinal cord injury. *Ann Palliat Med,* 9**,** 190-198.

HUANG, C., SUN, L., XIAO, C., YOU, W., SUN, L., WANG, S., ZHANG, Z. & LIU, S. 2023. Circular RNA METTL9 contributes to neuroinflammation following traumatic brain injury by complexing with astrocytic SND1. *J Neuroinflammation,* 20**,** 39.

HUANG, X. J., SU, G. J., WU, C. W., SHA, X. S., ZOU, J. F., LIU, X. S., LI, M. & HE, Y. 2022. Knockdown of rno_circRNA_009194 Improves Outcomes in Traumatic Brain Injury Rats through Inhibiting Voltage-Gated Sodium Channel Nav1.3. *J Neurotrauma,* 39**,** 196-210.

JIANG, Y. J., CAO, S. Q., GAO, L. B., WANG, Y. Y., ZHOU, B., HU, X., PU, Y., LI, Z. L., WANG, Q., XIAO, X., ZHAO, L., WANG, S., LIANG, W. B. & ZHANG, L. 2019. Circular Ribonucleic Acid Expression Profile in Mouse Cortex after Traumatic Brain Injury. *J Neurotrauma,* 36**,** 1018-1028.

LI, X., KANG, J., LV, H., LIU, R., CHEN, J., ZHANG, Y., ZHANG, Y., YU, G., ZHANG, X. & NING, B. 2021. CircPrkcsh, a circular RNA, contributes to the polarization of microglia towards the M1 phenotype induced by spinal cord injury and acts via the JNK/p38 MAPK pathway. *Faseb j,* 35**,** e22014.

LI, X., LOU, X., XU, S., DU, J. & WU, J. 2020. Hypoxia inducible factor-1 (HIF-1α) reduced inflammation in spinal cord injury via miR-380-3p/ NLRP3 by Circ 0001723. *Biol Res,* 53**,** 35.

LI, Y., WANG, B., SUN, W., KONG, C., LI, G., CHEN, X. & LU, S. 2022. Screening the immune-related circRNAs and genes in mice of spinal cord injury by RNA sequencing. *Front Immunol,* 13**,** 1060290.

LIU, C., GAO, R., TANG, Y., CHEN, H., ZHANG, X., SUN, Y., ZHAO, Q., LV, P., WANG, H., YE-LEHMANN, S., LIU, J. & CHEN, C. 2022. Identification of potential key circular RNAs related to cognitive impairment after chronic constriction injury of the sciatic nerve. *Front Neurosci,* 16**,** 925300.

LIU, Y., LIU, J. & LIU, B. 2020. Identification of Circular RNA Expression Profiles and their Implication in Spinal Cord Injury Rats at the Immediate Phase. *J Mol Neurosci,* 70**,** 1894-1905.

MAO, S., HUANG, T., CHEN, Y., SHEN, L., ZHOU, S., ZHANG, S. & YU, B. 2019a. Circ-Spidr enhances axon regeneration after peripheral nerve injury. *Cell Death Dis,* 10**,** 787.

MAO, S., ZHANG, S., ZHOU, S., HUANG, T., FENG, W., GU, X. & YU, B. 2019b. A Schwann cell-enriched circular RNA circ-Ankib1 regulates Schwann cell proliferation following peripheral nerve injury. *FASEB J,* 33**,** 12409-12424.

QI, J., WANG, T., ZHANG, Z., YIN, Z., LIU, Y., MA, L., PEI, S., DONG, Z. & HAN, G. 2022. Circ-Ctnnb1 Regulates Neuronal Injury in Spinal Cord Injury through the Wnt/β-Catenin Signaling Pathway. *Dev Neurosci,* 44**,** 131-141.

QIN, C., LIU, C. B., YANG, D. G., GAO, F., ZHANG, X., ZHANG, C., DU, L. J., YANG, M. L. & LI, J. J. 2018. Circular RNA Expression Alteration and Bioinformatics Analysis in Rats After Traumatic Spinal Cord Injury. *Front Mol Neurosci,* 11**,** 497.

QIN, C., LIU, Y., XU, P. P., ZHANG, X., TALIFU, Z., LIU, J. Y., JING, Y. L., BAI, F., ZHAO, L. X., YU, Y., GAO, F. & LI, J. J. 2022. Inhibition by rno-circRNA-013017 of the apoptosis of motor neurons in anterior horn and descending axonal degeneration in rats after traumatic spinal cord injury. *Front Neurosci,* 16**,** 1065897.

SIDDIQ, M. M., TORO, C. A., JOHNSON, N. P., HANSEN, J., XIONG, Y., MELLADO, W., TOLENTINO, R. E., JOHNSON, K., JAYARAMAN, G., SUHAIL, Z., HARLOW, L., DAI, J., BEAUMONT, K. G., SEBRA, R., WILLIS, D. E., CARDOZO, C. P. & IYENGAR, R. 2023. Spinal cord injury regulates circular RNA expression in axons. *Front Mol Neurosci,* 16**,** 1183315.

SOHN, E. J. & PARK, H. T. 2020. Differential expression of circular RNAs in the proximal and distal segments of the sciatic nerve after injury. *Neuroreport,* 31**,** 76-84.

SUN, J., LIAO, Z., LI, Z., LI, H., WU, Z., CHEN, C. & WANG, H. 2023. Down-regulation miR-146a-5p in Schwann cell-derived exosomes induced macrophage M1 polarization by impairing the inhibition on TRAF6/NF-kappaB pathway after peripheral nerve injury. *Exp Neurol,* 362**,** 114295.

SUN, Y., ZHOU, Y., SHI, X., MA, X., PENG, X., XIE, Y. & CAO, X. 2021. CircTYW1 serves as a sponge for microRNA-380 in accelerating neurological recovery following spinal cord injury via regulating FGF9. *Cell Cycle,* 20**,** 1828-1844.

TAN, W., MA, J., FU, J., WU, B., ZHU, Z., HUANG, X., DU, M., WU, C., BALAWI, E., ZHOU, Q., ZHANG, J. & LIAO, Z. 2024. Transcriptomic and bioinformatics analysis of the mechanism by which erythropoietin promotes recovery from traumatic brain injury in mice. *Neural Regen Res,* 19**,** 171-179.

TIAN, F., YANG, J. & XIA, R. 2022. Exosomes Secreted from circZFHX3-modified Mesenchymal Stem Cells Repaired Spinal Cord Injury Through mir-16-5p/IGF-1 in Mice. *Neurochem Res,* 47**,** 2076-2089.

TONG, D., ZHAO, Y., TANG, Y., MA, J., WANG, Z. & LI, C. 2021. Circ-Usp10 promotes microglial activation and induces neuronal death by targeting miRNA-152-5p/CD84. *Bioengineered,* 12**,** 10812-10822.

WANG, K., SU, X., SONG, Q., CHEN, Z., CHEN, H., HAN, Y., ZHU, C. & SHEN, H. 2023a. The circ_006573/miR-376b-3p Axis Advances Spinal Cord Functional Recovery after Injury by Modulating Vascular Regeneration. *Mol Neurobiol,* 60**,** 4983-4999.

WANG, W., HE, D., CHEN, J., ZHANG, Z., WANG, S., JIANG, Y. & WEI, J. 2021a. Circular RNA Plek promotes fibrogenic activation by regulating the miR-135b-5p/TGF-βR1 axis after spinal cord injury. *Aging (Albany NY),* 13**,** 13211-13224.

WANG, W., WANG, S., ZHANG, Z., LI, J., XIE, W., SU, Y., CHEN, J. & LIU, L. 2020. [Identification of potential traumatic spinal cord injury related circular RNA-microRNA networks by sequence analysis]. *Zhongguo Xiu Fu Chong Jian Wai Ke Za Zhi,* 34**,** 213-219.

WANG, W. Z., LI, J., LIU, L., ZHANG, Z. D., LI, M. X., LI, Q., MA, H. X., YANG, H. & HOU, X. L. 2021b. Role of circular RNA expression in the pathological progression after spinal cord injury. *Neural Regen Res,* 16**,** 2048-2055.

WANG, X., LI, W., HAO, M., YANG, Y. & XU, Y. 2023b. Hypoxia-treated umbilical mesenchymal stem cell alleviates spinal cord ischemia-reperfusion injury in SCI by circular RNA circOXNAD1/ miR-29a-3p/ FOXO3a axis. *Biochem Biophys Rep,* 34**,** 101458.

WENG, J., ZHANG, P., YIN, X. & JIANG, B. 2018. The Whole Transcriptome Involved in Denervated Muscle Atrophy Following Peripheral Nerve Injury. *Front Mol Neurosci,* 11**,** 69.

WU, C., DU, M., YU, R., CHENG, Y., WU, B., FU, J., TAN, W., ZHOU, Q., BALAWI, E. & LIAO, Z. B. 2022a. A novel mechanism linking ferroptosis and endoplasmic reticulum stress via the circPtpn14/miR-351-5p/5-LOX signaling in melatonin-mediated treatment of traumatic brain injury. *Free Radic Biol Med,* 178**,** 271-294.

WU, J., LI, X., WANG, Q., WANG, S., HE, W., WU, Q. & DONG, C. 2022b. LncRNA/miRNA/mRNA ceRNA network analysis in spinal cord injury rat with physical exercise therapy. *PeerJ,* 10**,** e13783.

WU, R., MAO, S., WANG, Y., ZHOU, S., LIU, Y., LIU, M., GU, X. & YU, B. 2019. Differential Circular RNA Expression Profiles Following Spinal Cord Injury in Rats: A Temporal and Experimental Analysis. *Front Neurosci,* 13**,** 1303.

XIAO, X., DENG, Q., ZENG, X., LAI, B. Q., MA, Y. H., LI, G., ZENG, Y. S. & DING, Y. 2022. Transcription Profiling of a Revealed the Potential Molecular Mechanism of Governor Vessel Electroacupuncture for Spinal Cord Injury in Rats. *Neurospine,* 19**,** 757-769.

XIE, B. S., WANG, Y. Q., LIN, Y., ZHAO, C. C., MAO, Q., FENG, J. F., CAO, J. Y., GAO, G. Y. & JIANG, J. Y. 2018. Circular RNA Expression Profiles Alter Significantly after Traumatic Brain Injury in Rats. *J Neurotrauma,* 35**,** 1659-1666.

XIE, L., ZHANG, M., LIU, Q., WEI, R., SUN, M., ZHANG, Q., HAO, L., XUE, Z., WANG, Q., YANG, L., WANG, H. & PAN, Z. 2023. Downregulation of ciRNA-Kat6b in dorsal spinal horn is required for neuropathic pain by regulating Kcnk1 in miRNA-26a-dependent manner. *CNS Neurosci Ther,* 29**,** 2955-2971.

XIN, Y., SONG, X. & GE, Q. 2021. Circular RNA SMEK1 promotes neuropathic pain in rats through targeting microRNA-216a-5p to mediate Thioredoxin Interacting Protein (TXNIP) expression. *Bioengineered,* 12**,** 5540-5551.

XIONG, W., WEI, M., ZHANG, L., WANG, J., LIU, F. & WANG, Z. 2022. Chronic constriction injury-induced changes in circular RNA expression profiling of the dorsal root ganglion in a rat model of neuropathic pain. *BMC Neurosci,* 23**,** 64.

XU, C., HU, H., YI, T., ZENG, X., HU, Y. & MA, J. 2022. Circular Ribonucleic Acid Expression Alteration in the Spinal Cord Tissue after Spinal Cord Injury in Rats. *Neuroimmunomodulation,* 29**,** 97-116.

XU, T., LI, Z. Y., LIU, M., ZHANG, S. B., DING, H. H., WU, J. Y., LIN, S. Y., LIU, J., WEI, J. Y., ZHANG, X. Q. & XIN, W. J. 2023. CircFhit Modulates GABAergic Synaptic Transmission via Regulating the Parental Gene Fhit Expression in the Spinal Dorsal Horn in a Rat Model of Neuropathic Pain. *Neurosci Bull,* 39**,** 947-961.

YAO, Y., WANG, J., HE, T., LI, H., HU, J., ZHENG, M., DING, Y., CHEN, Y. Y., SHEN, Y., WANG, L. L. & ZHU, Y. 2020. Microarray assay of circular RNAs reveals cicRNA.7079 as a new anti-apoptotic molecule in spinal cord injury in mice. *Brain Res Bull,* 164**,** 157-171.

YE, X., CHEN, Y., WANG, J., CHEN, J., YAO, Y., WANG, L. L. & ZHAO, F. 2021. Identification of Circular RNAs Related to Vascular Endothelial Proliferation, Migration, and Angiogenesis After Spinal Cord Injury Using Microarray Analysis in Female Mice. *Front Neurol,* 12**,** 666750.

YU, Y., XU, X., LIN, C. & LIU, R. 2023. Systematic identification of potential key microRNAs and circRNAs in the dorsal root ganglia of mice with sciatic nerve injury. *Front Mol Neurosci,* 16**,** 1119164.

ZAN, C., LI, J., LIN, F. & WANG, Z. 2022. Potential value of differentially expressed circular RNAs derived from circulating exosomes in the pathogenesis of rat spinal cord injury. *Front Neurosci,* 16**,** 1003628.

ZHANG, S. B., LIN, S. Y., LIU, M., LIU, C. C., DING, H. H., SUN, Y., MA, C., GUO, R. X., LV, Y. Y., WU, S. L., XU, T. & XIN, W. J. 2019. CircAnks1a in the spinal cord regulates hypersensitivity in a rodent model of neuropathic pain. *Nat Commun,* 10**,** 4119.

ZHANG, Y., GAO, T., LI, X., WEN, C. C., YAN, X. T., PENG, C. & XIAO, Y. 2021. Circ_0005075 targeting miR-151a-3p promotes neuropathic pain in CCI rats via inducing NOTCH2 expression. *Gene,* 767**,** 145079.

ZHAO, J., QI, X., BAI, J., GAO, X. & CHENG, L. 2020. A circRNA derived from linear HIPK3 relieves the neuronal cell apoptosis in spinal cord injury via ceRNA pattern. *Biochem Biophys Res Commun,* 528**,** 359-367.

ZHAO, R. T., ZHOU, J., DONG, X. L., BI, C. W., JIANG, R. C., DONG, J. F., TIAN, Y., YUAN, H. J. & ZHANG, J. N. 2018. Circular Ribonucleic Acid Expression Alteration in Exosomes from the Brain Extracellular Space after Traumatic Brain Injury in Mice. *J Neurotrauma,* 35**,** 2056-2066.

ZHENG, P., SHU, L., REN, D., KUANG, Z., ZHANG, Y. & WAN, J. 2022. circHtra1/miR-3960/GRB10 Axis Promotes Neuronal Loss and Immune Deficiency in Traumatic Brain Injury. *Oxid Med Cell Longev,* 2022**,** 3522492.

ZHOU, J., XIONG, Q., CHEN, H., YANG, C. & FAN, Y. 2017. Identification of the Spinal Expression Profile of Non-coding RNAs Involved in Neuropathic Pain Following Spared Nerve Injury by Sequence Analysis. *Front Mol Neurosci,* 10**,** 91.

ZHOU, Z. B., DU, D., CHEN, K. Z., DENG, L. F., NIU, Y. L. & ZHU, L. 2019. Differential Expression Profiles and Functional Predication of Circular Ribonucleic Acid in Traumatic Spinal Cord Injury of Rats. *J Neurotrauma,* 36**,** 2287-2297.

ZHOU, Z. B., NIU, Y. L., HUANG, G. X., LU, J. J., CHEN, A. & ZHU, L. 2018. Silencing of circRNA.2837 Plays a Protective Role in Sciatic Nerve Injury by Sponging the miR-34 Family via Regulating Neuronal Autophagy. *Mol Ther Nucleic Acids,* 12**,** 718-729.

ZU, C., LI, J., HE, X., JI, L. & LI, X. 2022. Identification of a circRNA-mediated comprehensive ceRNA network in spinal cord injury pathogenesis. *Exp Biol Med (Maywood),* 247**,** 931-944.
